# Synthesis of activated carbon composited with Egyptian black sand for enhanced adsorption performance toward methylene blue dye

**DOI:** 10.1038/s41598-023-28556-6

**Published:** 2023-03-14

**Authors:** Ahmed Salah Elkholy, Mohamed Saber Yahia, Mohamed Abdelsamei Elnwawy, Hosny Anwar Gomaa, Ahmed Shafek Elzaref

**Affiliations:** 1 Chemistry Department, Faculty of Science (Boys Campus), Al-Azhar University, Cairo, Egypt; 2 Main Defense Chemical Laboratories (M.D.C.L), Almaza, Cairo, Egypt

**Keywords:** Environmental chemistry, Materials chemistry, Physical chemistry, Surface chemistry, Chemical synthesis

## Abstract

The present study reports the feasibility of the synthesis of a novel porous composite adsorbent, prepared from olive stone activated carbon (OS400) and garnet (GA) mineral impregnations (referred to as OSMG). This composite (OSMG) was applied for its ability to adsorb a macromolecular organic dye. The composite’s structural characteristics were evaluated using various techniques such as Brunauer–Emmett–Teller (BET), Scanning Electron Microscopy equipped with Energy Dispersive X-ray spectroscopy (SEM–EDX), X-ray diffraction (XRD), and a Fourier transform infrared spectrometer (FT-IR). The specific surface area of the garnet (GA), (OS400), and (OSMG) were found to be 5.157 mg⋅g^−1^, 1489.598 mg⋅g^−1^, and 546.392 mg⋅g^−1^, respectively. The specific surface area of the new composite (OSMG) was promoted to enhance the adsorption of methylene blue (MB). Experiments were conducted under various conditions, including contact time, initial dye concentration, adsorbent dosage, pH, and temperatures. Data from these experiments were analyzed using several adsorption models including Langmuir, Freundlich, Temkin, and Dubinin-Radushkevich (D-R). The results indicated that, the adsorption fit best with the Freundlich model and that the adsorption process followed a pseudo-second-order kinetic mechanism. Additionally, the thermodynamic analysis indicated the adsorption of MB onto garnet(GA) adsorbents is endothermic, while the sorption onto (OS400) and (OSMG) is an exothermic and non-spontaneous process. The OSMG composite can be used for at least five cycles without significant loss of adsorptive performance, and can easily be separated from the water after treatment.

## Introduction

Anthropogenic activity and population expansion are frequently linked to the extent to which contaminants are present throughout the ecosystem^1^. Even minimal amounts of dye discharge in water can harm aquatic life, decrease light transmission, and negatively impact photosynthesis^2^. Worldwide reports of more than 100,000 commercial dyes are available^3^.

Today, everyone has virtually unlimited access to color, and more than one million tons are created per year^1,4^. One common dye used in these industries is methylene blue, which is water-soluble and can be harmful if ingested, inhaled, or contacted with the skin^5,6^. Exposure to methylene blue can cause eye irritation, methemoglobinemia, produce cyanosis, convulsions, tachycardia, dyspnea, irritate the skin, and, if swallowed, cause nausea, vomiting, and diarrhea and several other symptoms^7^. It is highly presence in effluents (highly visible at small amounts of dyes < 1 ppm)^6^.

The production of toxic dyes by certain industries can result in significant environmental pollution, particularly in the form of wastewater^8^. There are a variety of conventional technologies and strategies that have been used to remove these dyes from wastewater, including ion exchange, membrane technology, physicochemical methods, photochemical and photocatalytic processes, advanced oxidation, and biological techniques^9,10^.

The production of toxic dyes by certain industries can result in significant environmental pollution, particularly in the form of wastewater. There are a variety of conventional technologies and strategies that have been used to remove these dyes from wastewater, including ion exchange, membrane technology, physicochemical methods, photochemical and photocatalytic processes, advanced oxidation, and biological techniques.

Adsorption is a widely used and effective strategy for removing pollutants from impure wastewater^11,12^. The advantages of adsorption include high removal efficiency, simplicity, ease of application, and the ability to handle highly concentrated solutions^13,14^ Various adsorbent materials have been employed in the adsorption process, including clay minerals, nanomaterials, agricultural waste, and biological biomasses^15,16^. Additionally, researchers have used a number of specific adsorbents such as biochar, rice husk biochar, ZnO nanoparticles loaded rice husk biochar, biochar-MgO composite, Fe_3_O_4_/Clinoptilolite nanocomposite and composite adsorbent of Zn/Al layered double hydroxide and bagasse biochar for the treatment of wastewater^10^.

Activated carbon is one of the most often used adsorbents for water treatment because of its clear porous nature, efficacy, availability, higher capacity for adsorption, high surface area, a variety of porosity, and surface characteristics with a high degree of reactivity are also present^18,19^. Agricultural wastes are a good choice for making AC^20^. One of the widely accessible agricultural production byproducts is olive stones (OS)^21^. These wastes come from two main sources, namely the production of olive oil and table olives for human consumption^22^. Olive stone is composed of 5% ashes, 55% cellulose and hemicelluloses, 15% soluble compound, and 25% lignin^23^. With the food industry producing about 10 million tons annually^24,25^, and around 50–70 million tons of the Kraft lignin, a by-product of the pulp and paper industries, are generated worldwide^26,27^.

Carbon is produced and activated by various methods, chemically or physically, to improve some properties, and some of the physical methods by using radiation or microwaves^20,28^. The use of microwave technology to create AC from industrial agro-waste has been studied, and the results are encouraging^29^. They demonstrate that the process is economically viable because it is quick, high yield, selective heating, and can enhance the physical properties of materials such as high surface area, while also being environmentally friendly^30^. The electromagnetic waves which having frequencies between 300 MHz and 300 GHz are known as microwaves (wavelength between 1 and 1 mm)^31^. By means of ionic conduction and dipole rotation, microwave electromagnetic energy is converted into heat inside the particles resulting in rapid volumetric heating^32^. The large thermal gradient from the sample's inside to its surface enables the microwave-induced processes to proceed more quickly and effectively at a lower temperature, in less time, and with less energy^29^. Due to higher temperatures on the inside than the surface of the sample under these circumstances, the thermal gradient steadily diminishes from the center to the sample's surface, as a result of this temperature disparity, the low molecular weight components are quickly released, creating additional pores in the activated carbon^33^. On the other hand, oxides of intermediate metals, particularly manganese oxides, titanium oxide, and iron oxide have gained considerable interest due to their distinct physical and chemical properties^34^. Other commonly used sorbents to eliminate dyes and pollutants are sands^35^, and variable natural clay minerals such as: (Zeolites, Bentonite, and Diatomite, etc.) which are extensively applied in the adsorption process^36^. Natural sand was chosen as an adsorbent due to its quantity, efficiency, and nontoxicity. Several articles have employed various types of sand, including Sahara desert sand, sea sand, quartz sand, hematite sand, and hematite and titaniferous sand^37^.

Egyptian Black sand is a type of sand deposit that contains a high concentration of heavy metals of commercial relevance. These deposits can be found along the Mediterranean coast as a result of the mixing of Nile river water with Mediterranean seawater at the estuaries. Due to sea currents and waves, these sands were carried eastward along the coast, stretching for about 400 km from the cities of Rashid and Rafah^38,39^. The sand is black in color because of the high concentration of dark iron minerals, such as ilmenite, zircon, magnetite, rutile and garnet. These minerals are derived from metamorphic rocks found in the White Nile provenance^41^. This mineral has a common crystal structure and contains metal silicates of (Ca^2+^, Mg^2+^, Fe^2+^, Mn^2+^, Al^3+^, Fe^3+^, and Mn^3+^), and trace amounts of (V^3+^ and Cr^3+^). Nevertheless, natural garnets contain both (Ti^4+,6+^)^42^. Garnet can also be found in a variety of other rocks such as metamorphic, igneous and sedimentary rocks and can be formed from contact metamorphism, subsurface magma chambers, lava flows, and deep-source volcanic eruptions^40^.

In this study, the properties of an activated carbon adsorbent were improved by incorporating natural Egyptian garnet (GA) minerals. By adding GA, the adsorption efficiency was increased. Adsorption efficiency, cost-effectiveness, easy availability, compatibility, reusability, and enhanced mechanical, chemical, and thermal stability as well as reduced material consumption, and environmentally friendly process. The OS400 adsorbent was produced from olive stone and treated with garnet. In some preliminary experiments, the obtained data indicated that the OSMG adsorbent was the most convenient in achieving the best results.

This work aimed to use GA, OS400, and OSMG to eliminate the aqueous toxic organic compounds from water, an area that has not previously been studied. Additionally, the study also examined kinetic models, isotherm models, thermodynamics, and other parameters that impact the adsorption process.

## Experimental

### Materials

A stock solution of MB dye was prepared 1000 mg. L^−1^ by dissolving the required amount of dye powder in deionized water (Chemical formula, C_16_H_18_N_3_ClS, and molecular weight 319.85 g mol^−1^ supplied by Merck Co., Germany). Sodium Hydroxide (99% purity) and HCl acid 37% to justify the pH value, phosphoric acid (H_3_PO_4_ 85%) was applied to activate the adsorbent (OS400) produced from Olive Stone (local agro waste) via chemical method. The garnet (GA), type of black sand mineral has been collected from the region of Al-Burullus-Lake Coast, Kafr El-Sheikh governorate, Egypt.

### Preparation of Olive stone activated carbon (OS400)

The olive stone raw has been collected, Various pretreatment procedures were carried out, including washing and soaking well in deionized water, drying in an oven at 105 °C for 12 h, raw sizing/grinding, the particle size range of 1.5–2 mm, and sieving. The raw (OS) was soaked in aqueous phosphoric acid (H_3_PO_4_) solution (conc.85%) in a 1:3 (w/w) ratio and shaken for 2 h^43^. The mixture was dehydrated overnight at 105 °C in an oven followed by thermally activated in a laboratory furnace (NABERTHERM) at 400 °C with a constant heating rate of 10 °C /min for 2 h in the presence of inert nitrogen during the carbonization process with flow rate 150 cm^3^/min. The generated activated carbon (OS400) leave to cool down inside the furnace to room temperature then washed with 0.1 M HCl, then hot distilled water several times up to pH 6–7 for the filtrate solution. The final product dried at 105 °C for 24 h, then stored in a sealed container^20^.

### Synthesis of Olive stone activated carbon/Garnet (OSMG)

In order to create a composite of activated carbon and garnet, 0.5 g of the fine-grinding garnet (GA) was dispersed in 200 mL of distilled water with an ultrasonic agitator at a frequency of 40 kHz for 50 min. The presence of certain ions in natural garnets, such as Fe, Mn, Al, Ti, Ca, and Mg, which are dispersed in distilled water, may enhance the adsorbent's absorptive capabilities^34^. After the (GA) had completely dispersed in the water, about 20 ml from this solution had been put in a vessel (OMNI/XP1500, 100 ml) of Microwave model (MARS 5), with adding 10 g of activated carbon (OS400). The method parameters of the microwave were utilized at 15 min, Power 1200W, PSI 500, Temp 200˚C, and hold for 15 min. After the program is finished, the sample cools down in a microwave before opening the vessel. The sample (OSMG) eliminate from microwave vessel. Afterward, the samples were dried at 100˚C for 24 h^23,44^.

### Adsorption experiments

In this study, the elimination of methylene blue from aqueous solutions was investigated using chosen adsorbents. The effects of various parameters, including pH (3–9), initial concentration of the dye in the aqueous solution (1–5 mg/L), contact time (1–100 min), temperature (297 K to 323 K), and the adsorbent dose (0.25–1.5 g/L) were examined to determining the optimal adsorption conditions.

A batch technique was used to conduct the adsorption studies^45^. The sample was filtered with a Whattman syringe filter, 1.0 µm once the adsorption procedure was finished. The UV–vis spectrophotometer (HACH- DR/ 5000) set at 665 nm was used to measure the final concentrations of MB dye in the tested sample. The adsorption capacity (q_e_) and removal percentage yield (R %) were calculated using Eqs. 1 and 2, respectively^46^.

1 $${\mathrm{q}}_{\mathrm{e}}=\frac{\left({\mathrm{C}}_{\mathrm{i}}-{\mathrm{C}}_{\mathrm{e}}\right)\mathrm{V}}{\mathrm{W}\times 1000}$$

2 $$\left(\mathrm{R \%}\right)=\frac{{\mathrm{C}}_{\mathrm{i}}-{\mathrm{C}}_{\mathrm{e}}}{{\mathrm{C}}_{\mathrm{i}}}\times 100$$

where C_i_ (mg/l) and C_e_ (mg/l) are the initial and equilibrium concentration, respectively, V (L) is the MB volume, and W is the mass of adsorbent (g) Experimental.

### Adsorbent recycling

Experimental desorption procedure were executed at room temperature. A quantity of 100 mg of the prepared adsorbent material was initially combined with 10 ml of a solution of methylene blue (MB) at pH 7. After a period of 120 min of stirring, the adsorbent (OSMG) was separated and delicately washed with deionized water, before being placed into 0.1 molar nitric acid (HNO_3_) solution for 30 min. afterwards, The composite (OSMG) was again removed from the solution. The level of desorbed MB was then measured by UV–Vis spectrophotometer. The OSMG composite underwent five sequential cycles of adsorption–desorption to evaluate its regeneration efficiency and overall cost-effectiveness. The desorption efficiency was computed by utilizing Eq. (3):

3 $$\mathrm{Desorption \,Efficiency }(\mathrm{\%}) = \frac{C.V}{q.m}.100$$

where, C (mg.L^−1^) is the MB concentration in the desorption solution, V(L) is the desorption volume, q (mg.g^−1^) is the adsorbed amount of MB and m (g) is the mass of adsorbent used in the desorption tests.

### Characterization methods

The produced porous materials were physicochemically analyzed by using an X-ray diffractometer (X' Pert PRO PANalytical-Netherland device) at 25 °C with Cu Kα a monochromatic radiation source. The morphology images were assessed through Scanning Electron Microscopy, model "SEM, FEI inspects" with Energy-Dispersive X-ray Spectrometer (EDX), which was utilized to examine the microstructures of all samples both before and after MB dye loading. EDX detector was used to analyze the distribution of ions onto the adsorbent surfaces. The N_2_ adsorption–desorption profiles according to the Brauner-Emmet-Teller (BET by NOVA e-Series analyzer), using the classical helium void volume method. The main surface functional groups of (GA), (OS400), and (OSMG) were measured through Bruker Optik GmbH FT-IR analysis, utilized at room temperature in the spectral region of 400–4000 cm^-1^.

## Results and discussion

### Characterization of adsorbents

#### X-ray diffraction

**Figure 1:**
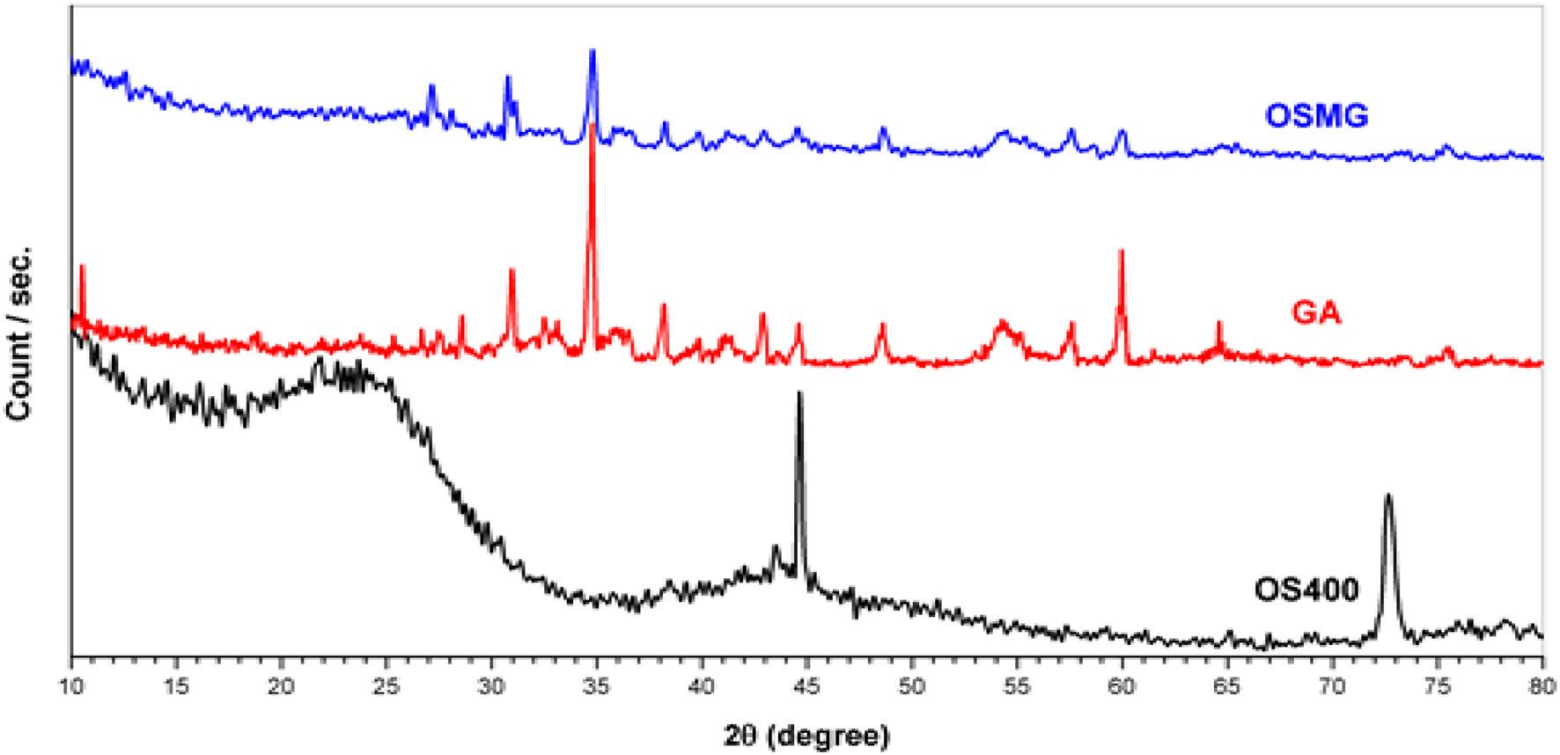
X- ray diffraction analysis of OS400, GA and OSMG.

Activated carbon (OS400), garnet (GA), and an AC/Garnet (OSMG) were all examined using XRD analysis in the range of 2ϴ from 10 to 80°. Figure 1. displays the X-ray diffraction patterns of samples. A broad peak with a greater intensity was formed for Olive stone Activated carbon (OS400) as a result of carbonization and chemical activation. This peak can be attributed to the development of cross-linked graphitic structures as a result of the bridging bonds between phosphate and polyphosphate molecules. This information suggests that the AC obtained has carbon-pore interfaces. Two diffraction peaks for 2θ were observed around 25° and 43° indicating the presence of a carbon structure in the samples, which correspond to the reflections (002) and (100/101) of the graphite structure, respectively^47^.

However, a sharp peak at 44.5° can be noticed in the case of AC (OS 400), indicating the presence of a crystalline component in the sample. On the other hand, this peak disappeared completely in the instance of the AC/Garnet (OSMG). This finding suggests that the crystalline component that would have been present on the carbon's surface was replaced by garnet metal ions^48^.

Furthermore, in the case of (GA), small sharp peaks appeared associated with some metals oxide. The peaks around 2θ ≈ 31˚and 35˚ may attributed to F_2_O_3_^49^, 2θ ≈ 28.3˚ and 47.5˚ for SiO_2_^50^, 2θ ≈ 32,47˚and 62˚ for Al_2_O_3_^51^, 2θ ≈ 28.3˚, 31˚, 38.1˚ and 54.5˚ for CaO^52^, 2θ ≈ 29˚, 37.5˚, 49˚, 57˚, 60˚, 65˚ For MnO_2_^53^, 2θ ≈ 60˚ MgO^54^, 2θ ≈ 38˚,48˚,55˚and 62˚ for TiO2^55^. Also, these metal oxide peaks appear with low intensity in the case of (OSMG), due to the few amount of these supported metal particles deposited on the surface of the porous using microwave support and the high dispersion of metal oxide species.

According to the previous sentence, there are many different types of metal ions dispersed throughout the OS400 surface, which causes a stronger connection between metal ions and the AC (OSMG) surface^56^.

#### SEM–EDX analysis

**Figure 2:**
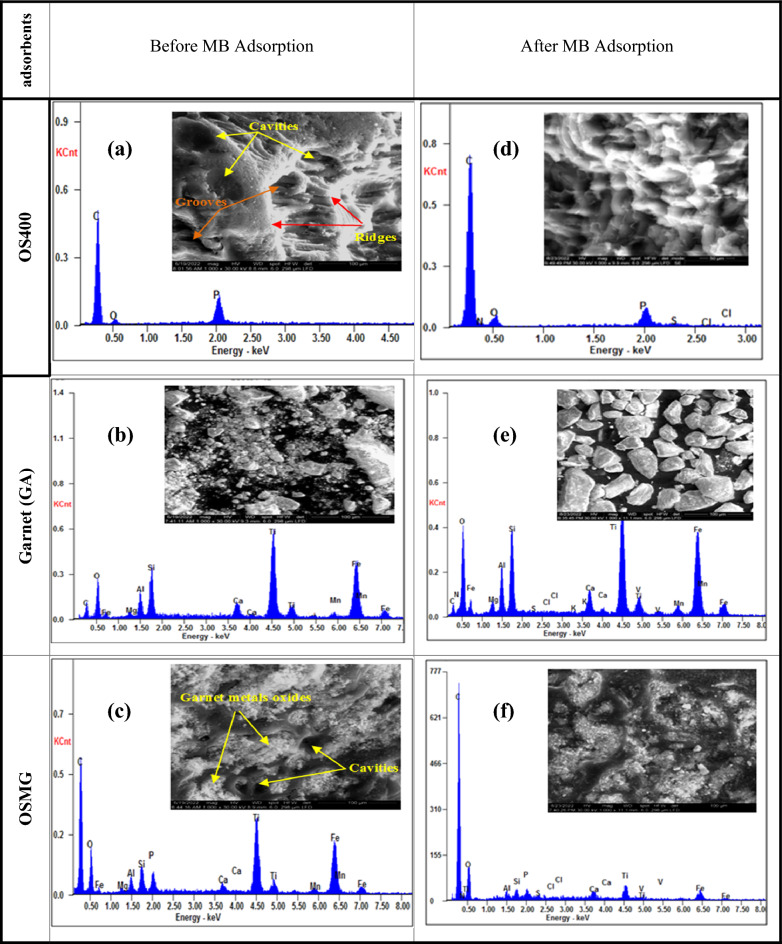
SEM images and EDX profiles for the adsorbents samples before (**a**, **b** and **c**), and after (**d**, **e** and **f**) the adsorption process of the MB dye.

SEM and EDX microchemical analyses were used to determine the detailed surface morphology of the activated carbon (OS400), Garnet (GA), and AC/Garnet adsorbents before and after the MB dye loading. The results are shown in Fig. 2 and Table 1.

**Table 1:** Chemical microanalysis of OS400, GA and OSMG before and after adsorption process.

Adsorbents		Elements (wt %)												
		C	O	Si	N	P	S	Cl	Fe	Al	Ca	Mg	Mn	Ti
OS400	Before adsorption	90.08	06.68	–	–	03.23	–	–	–	–	–	–	–	–
GA		2.75	25.80	09.69	–	–	–	–	27.29	04.33	01.99	01.22	02.30	24.61
OSMG		61.97	18.33	01.57	–	01.14	–	–	07.82	01.06	00.58	00.33	00.66	06.54
OS400	After adsorption	84.79	11.04	–	01.99	01.86	00.26	00.07	–	–	–	–	–	–
GA		08.90	29.51	10.33	01.58	–	00.13	00.11	22.13	04.97	02.40	01.73	01.96	16.00
OSMG		74.90	14.39	00.61	02.7	00.65	00.22	00.13	02.69	00.52	00.97	0.52	0.47	02.22

Figures 2a–c display the sample micrographs before the MB dye adsorption. The activated carbon (OS400) had a rough, compacted surface and non-uniformly sized porosity within the graphene sheets. The large and well-developed irregular cavities are clearly apparent as a dark spot on the activated carbon surface, there is a good possibility for dye to be trapped and adsorbed into this cavities^57^. Additionally, non-porous ridges, intra-layer grooves, and pockets Fig. 2a.

**Figure 3:**
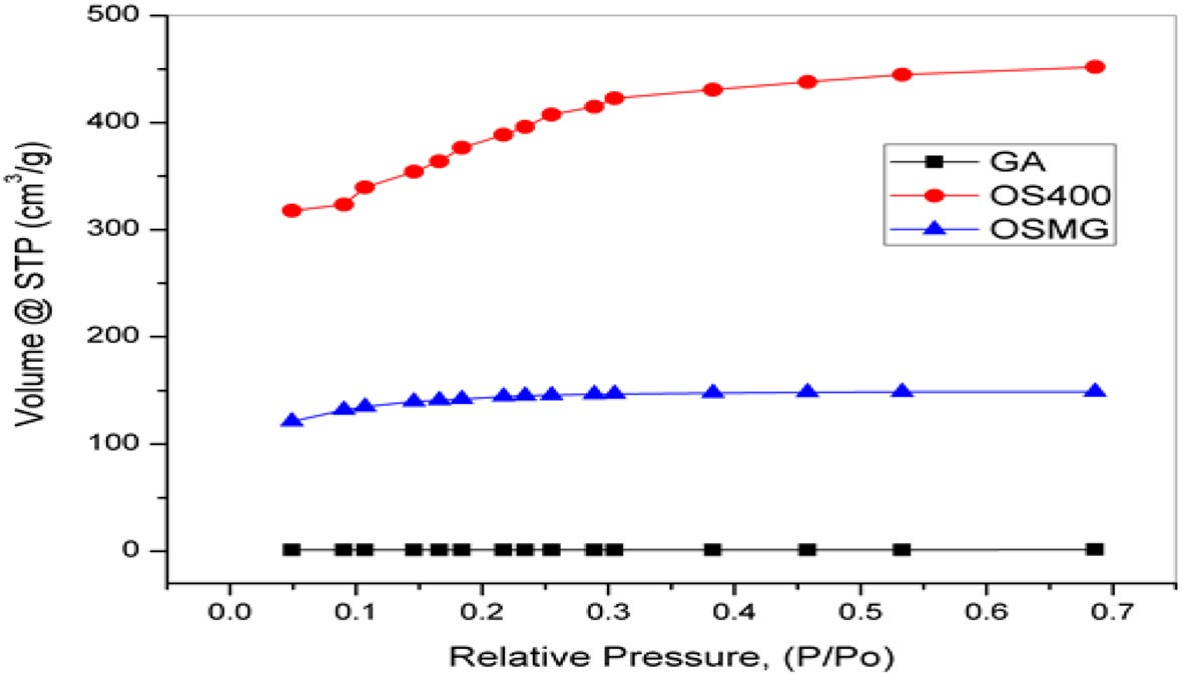
Nitrogen adsorption isotherms for adsorbents.

The high surface area and high porosity on the surface of activated carbon provide characteristic features, which can be used in the impregnation process; the BET analysis of samples illustrates the surface area and pore size distribution Fig. 3.

The garnet's (GA) morphology before MB uptake is depicted in Fig. 2b. The SEM images show that the granulometry of (GA) is different and the shapes of the particles were wide-ranging and irregular, rough surface with a number of pores, which can be effective in the adsorption process.

In the case of (OSMG), impregnating (OS400) with garnet while using microwaves causes the dispersion of garnet's metal oxides over the surface of activated carbon, as illustrated in Fig. 2c, this lead to filling cavities and clogging the cracks. The dispersion of metal oxide particles on the surface of (OSMG) carbon is evident in the SEM picture as white dots^58^.

On the other hand, Fig. 2d–f depicts the same materials after going through the MB dye adsorption process. After sorption, the majority of the pores were observed to be occupied by MB. The micrographs specified the dense texture of the sample's surface, also be smoother. Indicating that, the dye has densely and homogeneously adhered to the surface of adsorbents^59^.

The EDX chemical microanalysis before dye adsorption shows several elements in the composition of garnet, mainly composed of silicon besides other metals such as iron which gives the particles a black color, aluminum, manganese, titanium, and magnesium. The elemental compositions of garnet and other samples are given in Table 1.

The prepared materials OS400 contained only the elements that are typically found in activated carbon, with an average carbon content of about 90%. whereas the impregnated and modified carbon by microwave (OSMG) contained the same elements in addition to the mineral elements that are found in garnet as a result of the impregnation process such as Si, Fe, Al, Ca, Mg, Mn and Titanium.

On the other hand, after the adsorption of MB dye, the samples' EDX revealed additional components as N, S, and Cl. Table 1. and Fig. 2d–f. The methylene blue dye contains the components nitrogen, sulfur, and chlorine, proving that the adsorption process worked as intended. As opposed to (GA) and (OS400) adsorbents with a higher carbon content of 84.79%, it was found that sample (OSMG) having the least percentage of carbon content of 74.9% resulted in adsorbed the maximum amounts of the removed MB dye constituents (N, S, and Cl). Because the addition of garnet to carbon may have catalytic capabilities in the sorption process due to the iron and other metals present, including manganese, magnesium, aluminum, etc.^37,39^.

#### BET

NOVA e-Series analyzer was used to calculate the specific surface area (SE) of all prepared materials using nitrogen adsorption/desorption isotherms. The Brunauer Emmett Teller (BET) equation was used to get the surface area of the BET from the isotherm^60^. The measured surface area and the isotherms of N_2_ adsorption for (GA), (OS400), and (OSMG) are shown in Fig. 3 and Table 2.

**Table 2:** Pore structure parameters of garnet GA, Olive stones carbons OS400 and OSMG.

Adsorbents	*S*_*BET*_ (m^2^/g)	*V*_Micro_ (cc/g)	*V*_Meso_ (cc/g)	*r* (nm)	*V*_Total_ (cm^3^/g)	Aver _*Pore Size*_ (nm)
GA	5.157	0.001	0.005	11.018	0.313	4.881
OS400	1489.598	0.597	0.270	3.368	0.915	2.459
OSMG	546.392	0.218	0.004	3.341	0.851	1.693

The calculated BET for the garnet (GA) sample was 5.157 m^2^/g, which is a low value compared to the other samples. It was observed that the surface area of the sample (OSMG) lower than (OS400) as a result of using the microwave in the impregnation process with garnet ore, which led to filling and deposition in the activated carbon pores with garnet metal oxide^61^, especially Meso and Macropores, this interpretation is supported by SEM image.

#### FT-IR analysis

**Figure 4:**
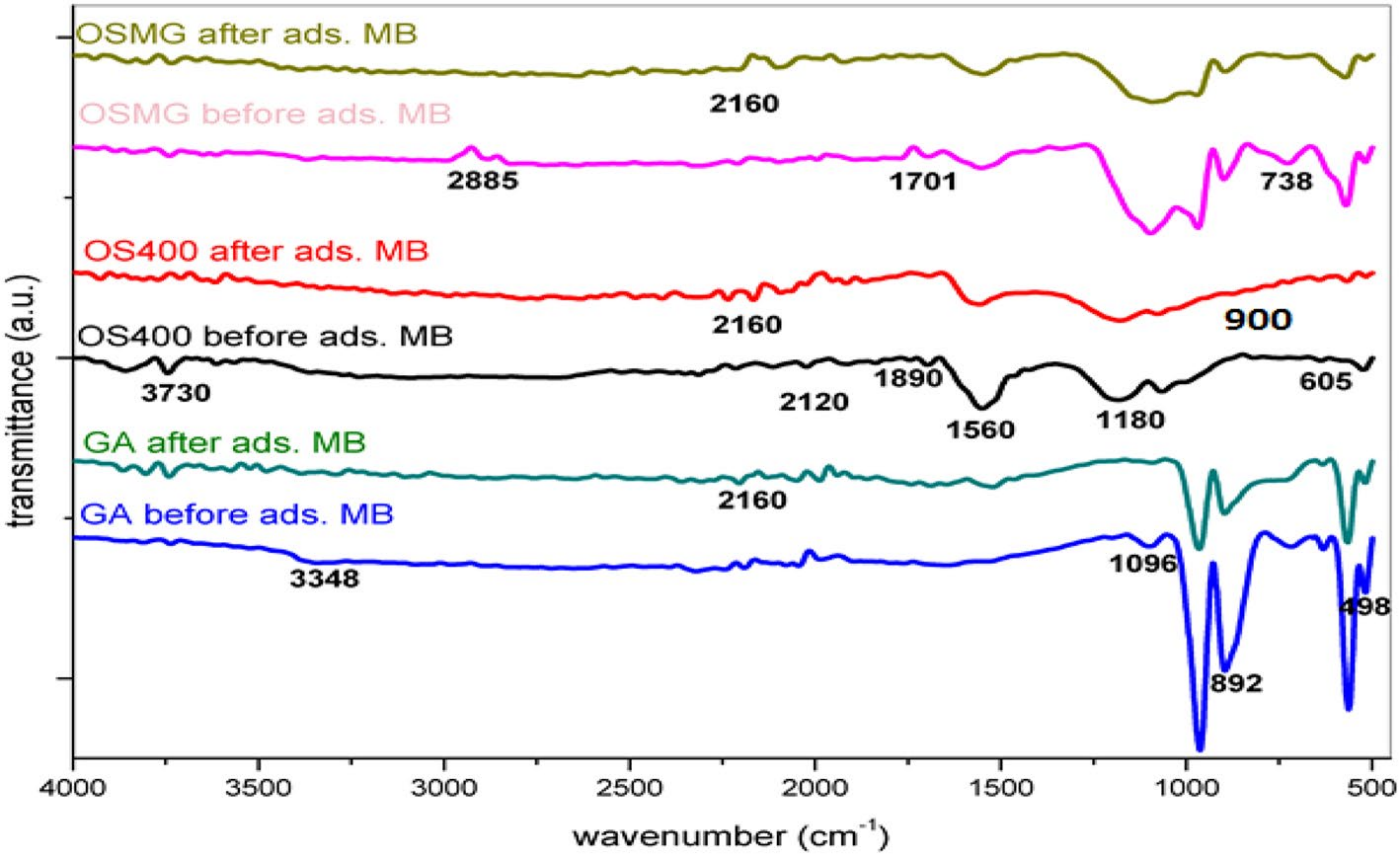
FT-IR spectra analysis of Garnet GA, OS400 and OSMG before and after adsorption of MB.

Figure 4 reveals the main peaks of (GA), (OS400), and the impregnated carbon assisted by microwave (OSMG) before and after uptake of MB dye. Various peaks were identified; all spectra show a transmittance peak at around 3700 cm^−1^. the weak sharp transmittance peak at 3730 cm^−1^ is present in spectra of carbons material activated with H_3_PO_4_ (OS400) and decreasing in carbons modified with Microwave (OSMG)^62^. The position and asymmetry of this peak indicate the presence of strong hydrogen -bonded OH group of alcohols and phenols and carboxylic acids^63^, also this peak can be assigned to the O–H stretching mode of hydroxyl groups, and the asymmetrical aliphatic C–H for methyl and methylene group^64^.

The (OSMG) absorption peak at 2900 cm^-1^ is attributed to the symmetric and asymmetric stress of the C–H of saturated aliphatic compounds due to the elongation of the CH groups^65^, those peak became visible after using a microwave.

The several minor peaks between 2200 and 1890 cm^−1^ are caused by the stretching of the C–O bond, whereas the small peak at around 1700 cm^−1^ is usually attributed to the vibrations of the C = O bond caused by the stretching of ketones, aldehydes, lactones, or carboxyl groups^66^. On the other hand, after the sorption of MB, a peak at around 2160 cm^−1^ was found, confirming the presence of the C = C stretching vibration of the quinoid structure^67^.

The spectra of the prepared activated carbons (OS400) and (OSMG) also show a strong peak at 1600–1560 cm^−1^ due to C–C vibrations in aromatic rings. A strong peak in the C–O stretching region at about 1180 cm^−1^ is often associated with oxidized carbons (wide band 1100–1300 cm^−1^) confirms the carbonyl band's assignment to an ester^66,68^. However, the peaks around 1190–1200 cm^−1^, albeit characteristic of phosphorous-containing functionalities, may also show hydrogen-bonded P–O and O–C stretching vibration in P–O–C (aromatic) and P–O in acidic phosphate esters^69^. The dehydration of cellulose in olive stone by phosphoric acid is analogous to that of alcohols, and at higher temperatures, phosphorous oxides act as Lewis acids and can create C–O–P bonds^70^.

The weak peaks at around 900 cm^−1^ of the two materials (OS400) and (OSMG) are due to the vibration group (CH) aromatic amine group (N–H)^71^. There are some peaks observed in (GA) and (OSMG) materials and that do not exist in activated carbon (OS400), these peaks in the range of 542 − 790 cm^−1^ in (GA) and (OSMG) samples can be due to the tensile vibrations of Si–O–Si, Si–O–Al, and Si–O–Mg and the bending vibrations of Si–O, iron oxides at 700–600 cm^−1^ are in the structure of garnet (GA) and also, appears in (OSMG) due to the impregnation process of activated carbon assisted with microwave^72^.

### Effect of adsorbent dosage

**Figure 5:**
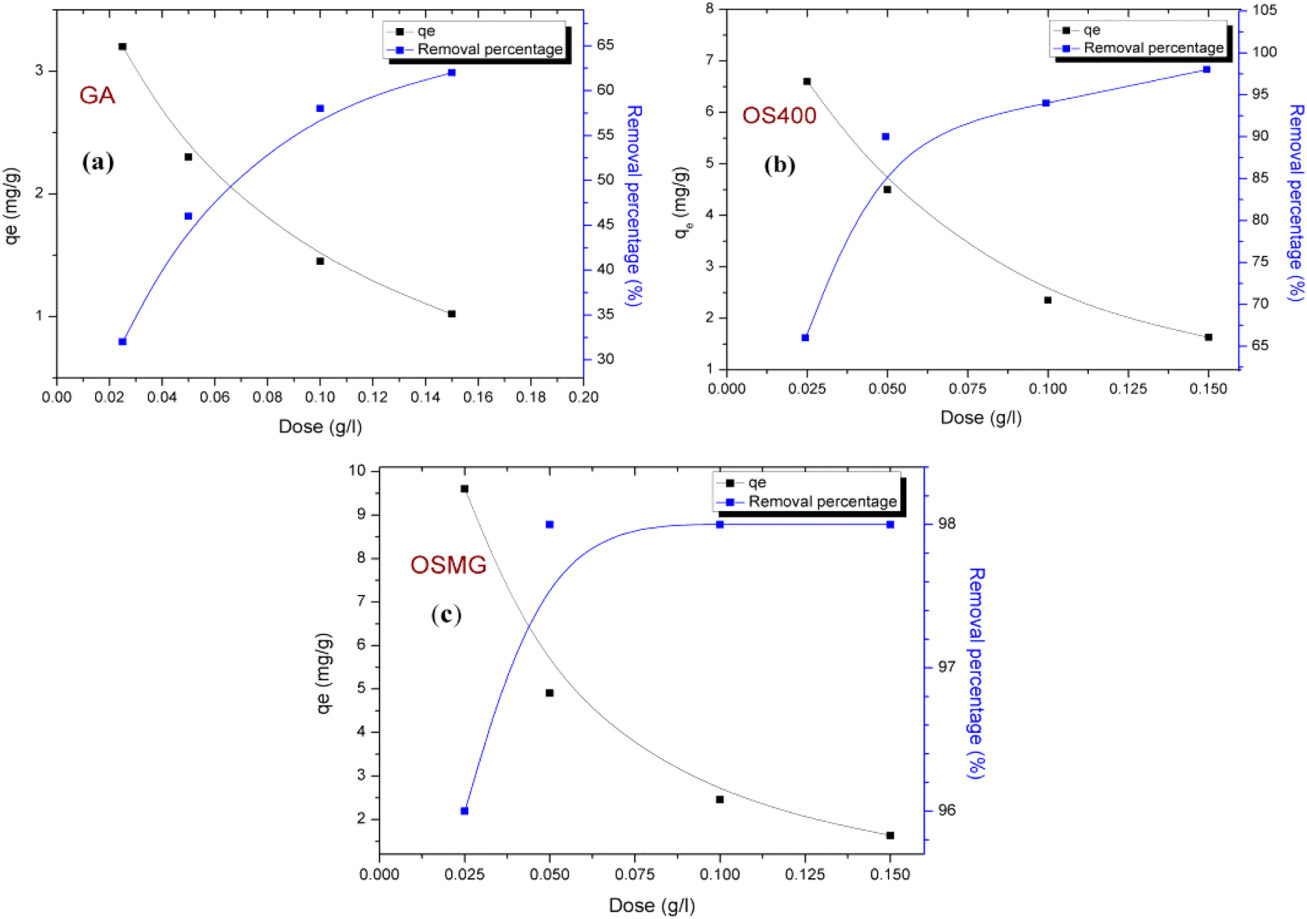
The adsorption capacity (q_e_ mg.g^−1^) and the adsorption efficiency (R %) of GA, OS400 and OSMG (**a**, **b** and **c**) for the adsorbed MB.

As the data are shown in Table 3 and Fig. 5 the increasing of the adsorbent dosage from 0.025 to 0.15 g/L, the uptake percentage improves from 0 to 100 min to 62%, 97%, and 98% for (GA), (OS400), and (OSMG), respectively. This is owing to the great availability of a large number of active centers for dye adsorption on the surface of adsorbents^73^. Meanwhile, the amount of MB adsorbed, q_e_,_cal_ (mg g^−1^) at equilibrium decreases with increasing adsorbent dose, this regressed on is attributed to the high number of unsaturated adsorption sites. It is noteworthy that (OSMG) material achieved the highest removal rate of 98% and reached equilibrium very quickly after only 20 min compared to the other samples (GA) and (OS400), which makes it superior in the sorption process. However, no increase in the removal % of dye adsorption was observed with increasing the adsorbent dosage above 0.05 g/L for (OSMG).

**Table 3:** Effect of adsorbent dose on R(%) and q_e_ (mg. g^−1^) of MB dye after 100 min.

Adsorbents	0.025 g/L		0.05 g/ L		0.1 g/ L		0.15 g/ L	
	R%	q_e_ (mg g^-1^)	R%	q_e_ (mg g^-1^)	R%	q_e_ (mg g^-1^)	R%	q_e_ (mg g^-1^)
GA	32.00	3.20	46.00	2.30	58.00	1.45	62.00	1.03
OS400	66.00	6.60	90.00	4.50	94.00	2.35	97.00	1.6
OSMG	96.00	9.60	98.00	4.90	98.00	2.45	98.00	1.63

### Effect of initial concentration

In this study, the effects of various initial MB dye concentrations ranging from 1 to 5 mg/l were performed on the adsorbents in order to determine the rate of adsorption capacity and efficacy. To carry out this experiment, a fixed dose (0.025 g) of the adsorbents (GA), (OS400), and (OSMG) were exposed to a 50 ml treatment with 5 ppm MB dye solution at pH 7. The contact time was 100 min, accompanied by a slight mechanical shaking at room temperature. Table 4 summarizes the results collected.

**Table 4:** Effect of initial concentration on (R%) and qe (mg. g^-1^) of MB dye after 100 min.

Adsorbents	1 ppm		2 ppm		3 ppm		4 ppm		5 ppm	
	R%	q_e_ (mg g^−1^)	R%	q_e_ (mg g^−1^)	R%	q_e_ (mg g^−1^)	R%	q_e_ (mg g^−1^)	R%	q_e_ (mg g^−1^)
GA	60.0	1.20	50.0	2.0	40.0	2.40	35.0	2.80	32.0	3.20
OS400	80.0	1.60	75.0	3.0	70.0	4.20	67.5	5.40	66.0	6.60
OSMG	90.0	1.80	95.0	3.8	96.6	5.80	95.0	3.80	96.0	9.60

This study’s findings demonstrate that the adsorption process is strongly influenced by the dye's initial concentration in solution; for example, in the case of (GA) and (OS400), MB removal percentages decrease as initial concentrations increase. i.e. for lower concentrations, the R % of dye uptake was higher than the higher concentrations, and the outcomes can be compared to those of other similar studies^74,75^. Because there are more active sites on the adsorbent surface than there are dye molecules at lower concentrations, the interactions between dye molecules and the adsorbent are higher. As a result, the percentage of removal is further increased^76^. On the other hand, the adsorption capacity is steadily raised^77^.

**Figure 6:**
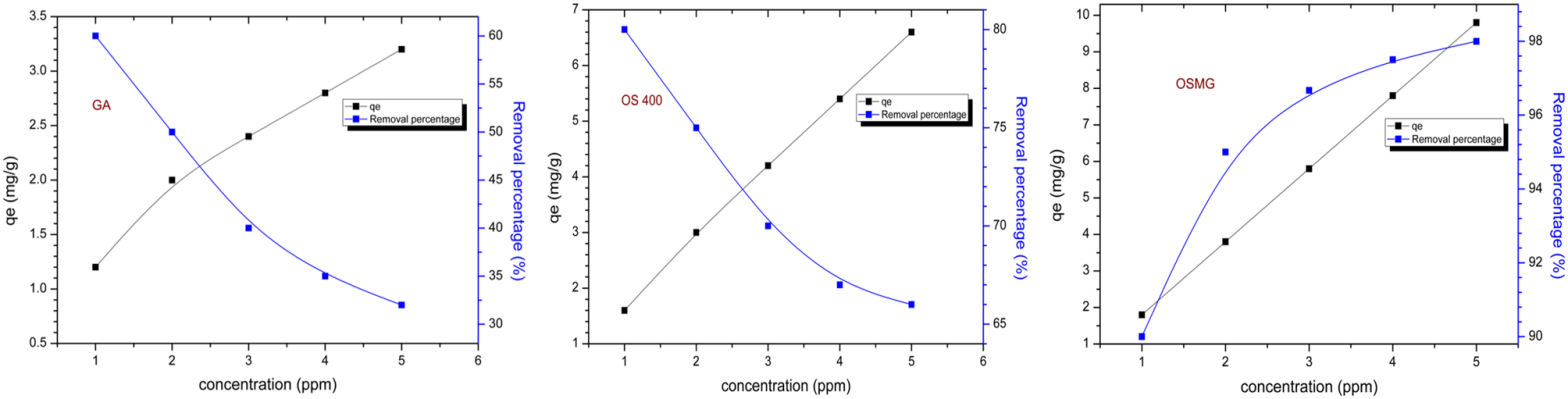
Effect of different concentrations of MB dye adsorbed onto GA, OS400 and OSMG.

Furthermore, in the case of (OSMG), the results of the adsorption process showed different behavior. However, the sorption percentages increase as initial concentrations increase, as shown in Fig. 6. This action may be due to the availability of more favorable sites for ion exchange between the MB ions solution and the ions on the surface of (OSMG)^78^.

The data results show that the adsorption efficiency of (OSMG) sample at the highest concentration (5 ppm) was superior compared with other samples, which proves the high performance of the prepared composite (OSMG). Generally, the results of THE sorption process showed a favorable effect for the activated carbon species, possibly due to the larger surface area for OS400 and (OSMG) versions than garnet (GA)^79^. Also, the enhanced effect of various metals oxide on the surface of (OSMG) is due to the impregnation process.

### Effect of pH

The pH of the solution is an important control parameter for the adsorption process. The positive or negative charges on the adsorbent surface may be increased, decreased, or neutralized as a result of pH variation^7^. The adsorption of adsorbate molecules on the surface of adsorbents can be improved or hampered by changes in surface negativity or positivity, respectively.

**Figure 7:**
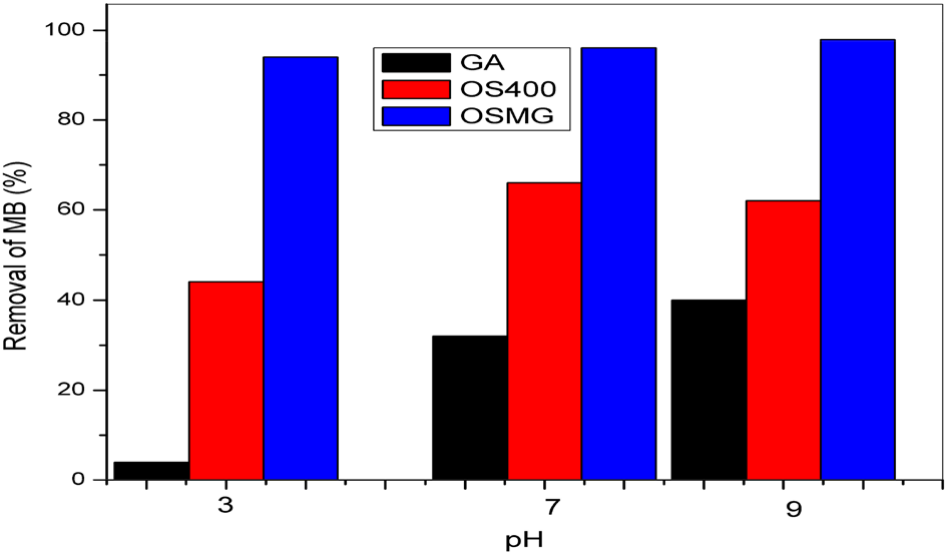
Effect of solution pH on the MB adsorption (adsorbent dose: 0.025 g. L^−1^, dye concentration: 5 mg.L^−1^).

In order to study the effect of the pH parameter, the experiments were carried out at pH values of 3, 7, and 9, the value of pH was adjusted by adding either 0.1 N HCl or NaOH solutions to investigate the effect of pH value on MB adsorption, with the initial concentration of MB dye 5 mg/L, adsorbent dose 0.025 g and 200 rpm of shaking at room temperature. The effect of pH on the adsorption of MB onto (GA), (OS400), and (OSMG) is shown in Fig. 7.

The removal percentage (R%) values increased as the pH value increased, indicating that MB adsorption on (GA) and (OSMG) preferred a basic environment. The maximum adsorption was observed for both adsorbents at pH 9, where it was about 40% for (GA) and 98.0% for (OSMG), respectively. At higher pH, the surface of (GA) and (OSMG) may become negatively charged due to there is an increase in the hydroxyl ions, which improved the positively charged cations of dye via electrostatic attraction force between cationic dye and the adsorbents leading to an increase in sorption^67,80^.

However, the uptake results of MB onto (OS400) revealed that there was no benefit at pH values higher than pH7 and that maximum uptake was observed at pH 7 with the removal of 66%, a similar study has been previously observed^81,82^.

### Effect of temperature

**Figure 8:**
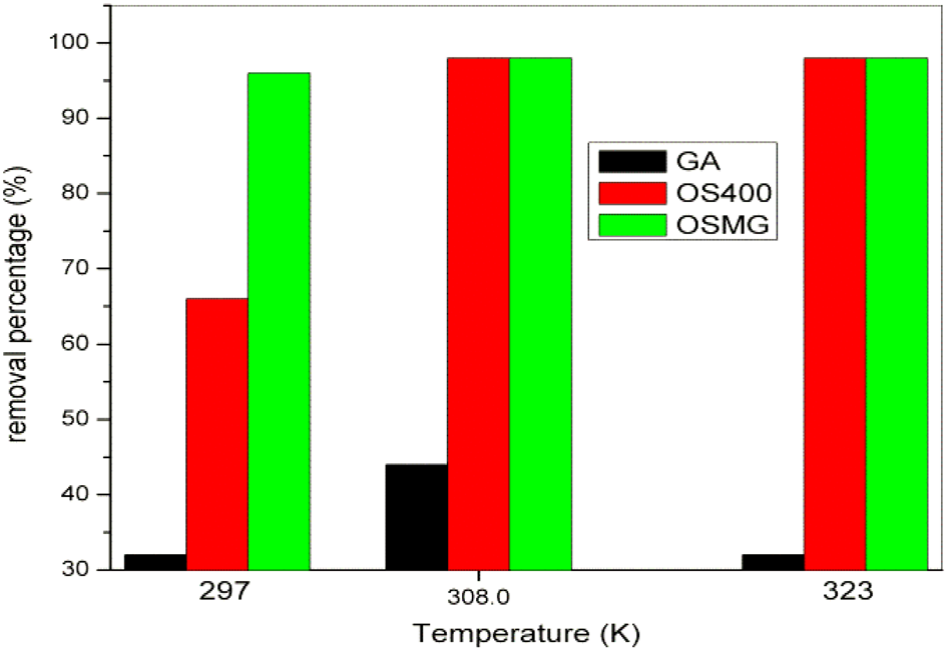
Effect of different temperatures on the removal percentage of MB adsorbed onto GA, OS400 and OSMG.

The effect of temperature is a significant physio-chemical process parameter, it plays an important role in the adsorption process and can change the sorption capacity of the adsorbent^83,84^. Figure 8 illustrates how temperature affects the adsorption of methylene blue onto activated carbon species (GA), (OS400) and (OSMG).

The results show that with increasing temperature from 297 to 323 K, the removal percentage of MB increased from 66 to 98% by (OS400) and maximum elimination by (OSMG) also 98% respectively, it is observed that there is no change in the removal % of MB of both adsorbent (OS400) and (OSMG) after 308 K and be fixed at 98%, so the optimum temperature in this case observed at 308 K. The increasing removal efficiency of dye at a high-temperature rate may be attributed to the solution's decreased viscosity, as result in the rate of diffusion of the dye molecules through the external boundary layer and inside pores of the adsorbent particle increases as the temperature rises, also increasing mobility of the dye molecules^85^.

On the other hand, the higher adsorption percentage of MB onto garnet (GA) adsorbent was 44% at 308 K, and this percentage slowly decreased by raising the temperature to 323 K to be 32%^86^. This behavior might be caused by the MB dye molecule's motions and colliding more quickly when rising temperature and may decrease the adsorptive forces between the dye species and the active sites on the adsorbent surface which reduces the removal efficiency^86,87^.

### Effect of contact time

The effect of contact time was examined by keeping the other variables constant. The removal of MB dye was measured by contacting 0.025 g of the adsorbents (GA), (OS400), and (OSMG) with 50 ml of a 5 mg l^-1^ MB solution at pH 7, the mixture was shaken in a mechanical shaker at 200 rpm with various contact times of (10–100 min).

**Figure 9:**
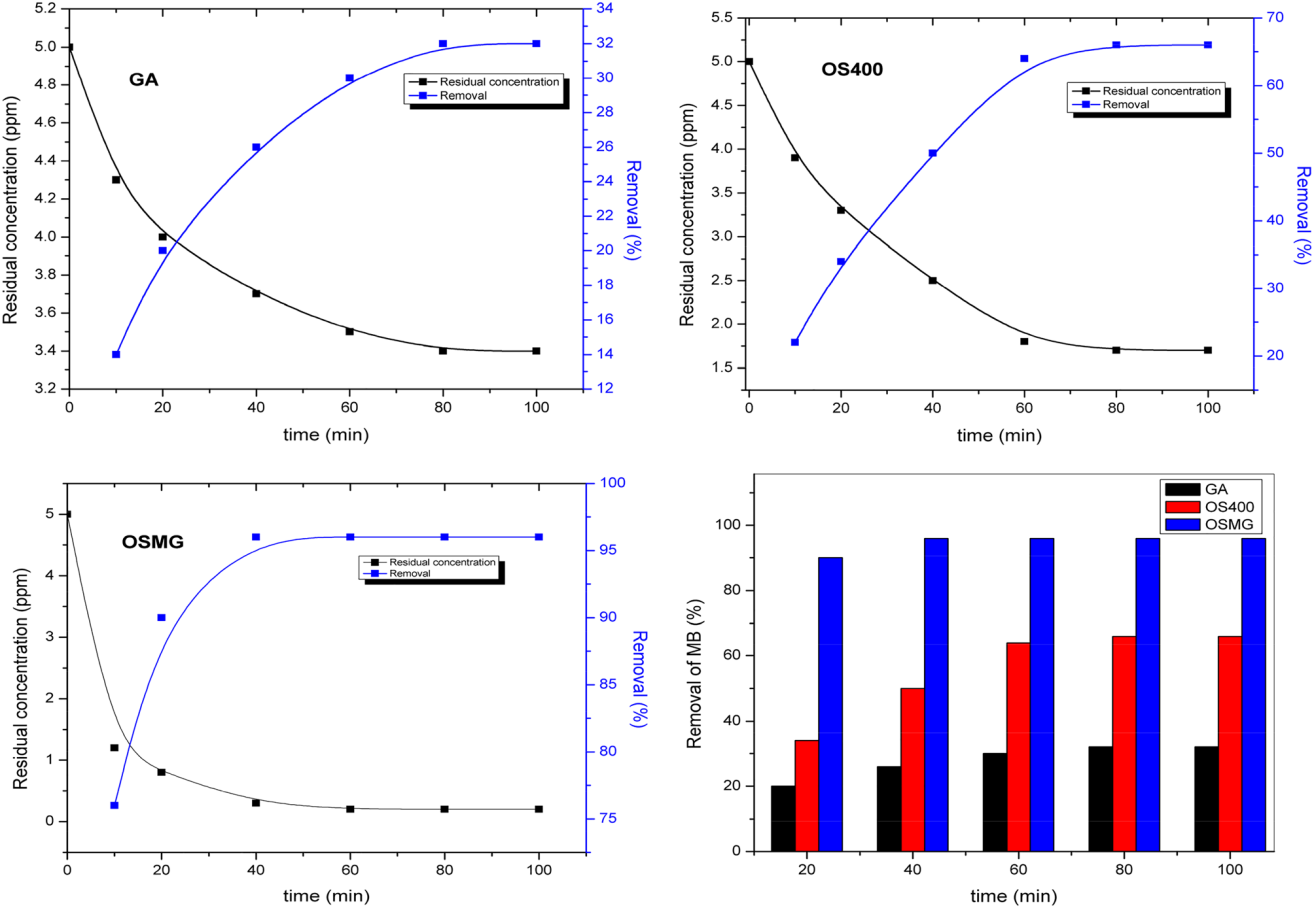
Effect of contact time on the removal percentage of MB dye.

Figure 9 shows that the rate of MB dye sorption increases with an increase in contact time^82^. It’s observed that the process of adsorption of MB achieves equilibrium after 80 min on (GA), 65 min on (OS400), and 40 min on (OSMG). However, the reaction rate was fast in the first 20 min in the case of (OSMG), with a clearance percentage of 90%, indicating that the rate of dye adsorption onto (OSMG) was faster than other adsorbents (GA) and (OS400). Figure 9 represents that, the maximum uptake of (5 ppm) of MB dyes by (OSMG) and (OS400) were 96% and 66% respectively.

### Adsorption isotherm

Equilibrium adsorption isotherms models are crucial to the design of any adsorption system. In order to determine the kind of adsorption between the adsorbents and MB dye solution^88^. In this study, the equilibrium sorption data were analyzed using linearized versions of Freundlich, Langmuir, Temkin, and D-R isotherms Eqs. (4–10) ^87,89,90^ respectively.

4 $$\mathrm{ln}{q}_{e}=\mathrm{ln}{K}_{F}+\frac{1}{n}\mathrm{ln}{C}_{e}$$

where K_*F*_ (mg.g^−1^) is the Freundlich model's adsorption capacity and *n* (dimensionless) is the adsorption intensity and refers to the heterogeneity of the system, K_*F*_ and *n* are considered the Freundlich isotherm constants.

5 $$\frac{{\mathrm{C}}_{\mathrm{e}}}{{\mathrm{q}}_{\mathrm{e}}}=\frac{1}{{\mathrm{KLQ}}_{\mathrm{max}}}+\frac{{\mathrm{C}}_{\mathrm{e}}}{{\mathrm{Q}}_{\mathrm{max}}}$$

6 $${\mathrm{R}}_{\mathrm{L}}=\frac{1}{1+{\mathrm{KLC}}_{\mathrm{o}}}$$

where ${C}_{e}$ is the equilibrium concentration of adsorbate (mg L^−1^), ${q}_{e}$ is the amount of sorbate at equilibrium (mmol g^−1^), Q_max_ (mg.g) represents the maximum monolayer adsorption capacity and K_L_ (L.mg^-1^) the Langmuir constant. Equation 5 also gives the separation factor (R_L_) which can be used to characterize the adsorption favorability on the adsorbent surface and C_0_ is the initial dye concentration.

7 $${\mathrm{q}}_{\mathrm{e}}=\frac{\mathrm{RT}}{{\mathrm{B}}_{\mathrm{T}}}ln({\mathrm{A}}_{\mathrm{T}} {\mathrm{C}}_{\mathrm{e}})$$

where B_T_ (J/mol)—Temkin isotherm constant, A_T_ (L/g)—Temkin isotherm equilibrium binding constant and R is the universal gas constant (8.314 J/mol/K) and T is the temperature K.

8 $$ln{\mathrm{ q}}_{\mathrm{e}}={\mathrm{lnq}}_{d}-\beta \varepsilon 2$$

9 $$\upvarepsilon =\mathrm{RTln}\left[1+\frac{1}{{\mathrm{C}}_{\mathrm{e}}}\right]$$

10 $$E=\frac{1}{\surd 2\upbeta }$$

where q_d_ (mg/g) D-R constant, β-the constant related to free energy, ε Polanyi potential and E (kJ/mol) the mean free energy.

**Figure 10:**
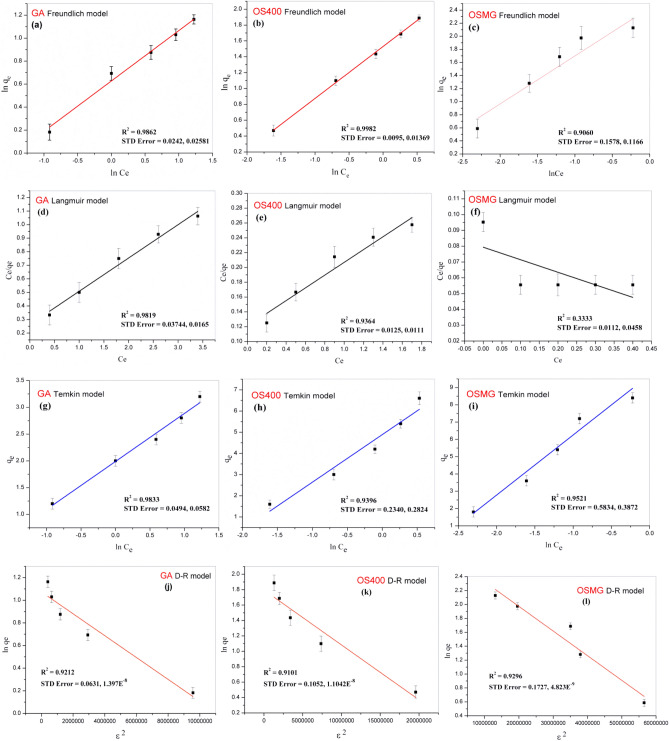
The linear plots of (**a**, **b** and **c**) Freundlich, (d, e and f) Langmuir, (**g**, **h** and **i**) Temkin, (**j**, **k** and **L**) D-R isotherm models for GA, OS400 and OSMG adsorbents, respectively.

The linear isotherm models for methylene blue (MB) adsorption are presented in Fig. 10, and the parameters for the Langmuir, Freundlich, Temkin and D-R models are listed in Table 5. The findings indicate that the Freundlich model has a better correlation coefficient (R^2^) that are closer to 1 compared to the Langmuir model, implying a multilayer adsorption mechanism for non-uniform surfaces with active sites distributed exponentially.

**Table 5:** Adsorption isotherm modeling for MB adsorption onto GA, OS400 and OSMG.

Models	Parameters	Adsorption of MB onto Adsorbent		
		GA	OS400	OSMG
Freundlich	*1/n*	0.4449	0.6524	0.7696
	*K*_*f*_ (mg/g)	0.6240	1.5261	2.4943
	*R*^*2*^	0.9862	0.9983	0.9061
Langmuir	*Q*_*max*_ (mg/g)	41.041	111.303	141.599
	*K*_*L*_(L/mg)	0.953	0.741	1.000
	*R*_*L*_	0.173- 0.512	0.213–0.575	0.167–0.50
	*R*^*2*^	0.9820	0.9364	0.3333
Temkin	*A*_*T*_ (L/g)	2.218	2.174	2.801
	*B*_*T*_ (J/mol)	0.896	2.247	3.478
	*R*^*2*^	0.9833	0.9396	0.9521
D-R	q_*d*_ (mg/g)	10.0736	25.0544	82.6799
	β (mol^2^/kJ^2^)	9.66 × 10^–8^	7.12 × 10^–8^	3.54 × 10^–9^
	*E* (kJ/mol)	2.274	2.650	11.884
	*R*^*2*^	0.9212	0.9101	0.9296

Also, the highest adsorption capacities for MB were observed for GA, OS400, and OSMG with values of 41.014, 111.30, and 141.599 mg/g, respectively. The R_L_ and 1/n values indicate that the adsorption process of MB using all adsorbents is favorable, and Table 5 shows R_L_ < 1, that confirms the suitability of the adsorbent materials for this kind of dye elimination^67^.

The Freundlich, Langmuir, Temkin and D-R isotherm models are used to describe the adsorption process by providing a theoretical understanding of the adsorbent-adsorbate interaction, each model helps to characterize the adsorption process by providing different parameters such as adsorption capacity, binding energy and heat of adsorption, and the nature of the adsorption process, whether it's a physisorption or chemical adsorption^90^.

The Temkin isotherm model suggests that the heat of adsorption of all molecules in the layer decreases linearly with increasing surface coverage. The Temkin model's binding energy indicates the electrostatic force of attraction between the adsorbent and the adsorbate. According to Table 5, the Temkin model has R^2^ values between 0.983 and 0.939, and the isotherm equilibrium binding constants (A_T_) are between 2.8 and 2.2 (L/g). Furthermore, the constants (B_T_) for the heat of methylene blue (MB) adsorption onto adsorbents GA, OS400, and OSMG were calculated to be 0.896, 2.247, and 3.478 J/mol respectively. Except for GA adsorbent, all B_T_ values obtained from the Temkin model are greater than 1, indicating that electrostatic interaction occurs and the heterogeneity of the pores on OS400 and OSMG surfaces plays a significant role in MB adsorption.

The Dubinin-Radushkevich (D-R) isotherm model is used to understand the mechanism of adsorption by considering the Gaussian energy distribution on the surface of the adsorbent. Table 5 displays the parameters of the D-R model with correlation coefficients of R^2^ values ranging between 0.921 and 0.929. The mean free energy (E) obtained from the D-R plots provides a glimpse into the type of adsorption process. A low E value below 8 kJ/mol is an indication of physisorption, while a range between 8 and 16 kJ/mol suggests chemisorption.. In this study, the OSMG adsorbent had an E value of 11.884 kJ/mol, signifying that the adsorption process is chemical in nature.

Based on the data in Table 5, it can be inferred that the OSMG material has a higher adsorption capacity for methylene blue (MB) than GA and OS400 materials, as it has high *q*_*max*_*, K*_*f*_ and *n* constant values. The adsorption capacities (q_max_) of the prepared composites were also compared with other adsorbents, as shown in Table 6.

**Table 6:** Comparison of several MB adsorption capacities (q_*max*_) reported in the literature**.**

Adsorbents	q_*max*_ (mg/g)	Reference
Olive stones activated carbon Composited With Garnet Black Sand (OSMG)	141.599	Present study
Olive stones activated carbon (OS400)	111.303	
Garnet black sand (GA)	41.041	
Activated cypress tree cone (H_3_PO_4_-CTC)	4.50	^93^
ZnCl_2_-RP	44.8	^94^
Olive stones activated carbon (OSAC)	16.12	^82^
Tamarind Seeds (AC)	1.42	^75^
Natural sand (Agdez, Assa)	12.15 and 16.00	^37^
Activated carbon from the banana stem (ACBS)	101.01	^74^
Biosorbents Based on Biopolymers from Natural Sources	188.679	^6^
Watermelon rind activated carbon (WMR-AC)	284	^44^
Activated carbon was developed from *Ficus carica* bast (FCBAC)	47.62	^95^

### Kinetic study

The adsorption kinetics models explain the dynamics of the MB dye adsorption reaction process^96^. The rate constant for the adsorption of MB onto (GA), (OS400), and (OSMG) was calculated using pseudo-first order (PFO), pseudo-second order (PSO) and intra-particle diffusion (IPD) models expressed by Eq. (11–14) ^11,97^. As shown in Table 7.

**Table 7:** Kinetics parameters of models on GA, OS400 and OSMG adsorbents.

Adsorbent	Conc.(ppm)	Pseudo first-order model			Pseudo second-order model				Intraparticle diffusion model		
		q_*e,1,cal*_ (mg/g)	*K*_*1*_ (min^-1^)	*R*^*2*^	q_*e*,*2*,*cal*_ (mg/g)	K_2_ (g/mg min)	h (mg/g min)	R^2^	K_*int*_ mg/g min^-0.5^	*C* (mg/g)	*R*^*2*^
GA	*5*	1.098	0.042	0.985	3.855	0.0144	0.215	0.996	0.3223	0.372	0.939
	*4*	1.036	0.041	0.986	3.590	0.0114	0.147	0.993	0.2939	0.192	0.956
	*3*	0.939	0.048	0.991	3.066	0.0144	0.136	0.983	0.2556	0.184	0.924
	*2*	0.827	0.058	0.980	2.546	0.0185	0.120	0.961	0.2146	0.177	0.879
	*1*	0.249	0.089	0.966	1.292	0.1347	0.225	0.991	0.1112	0.299	0.736
OS400	*5*	2.161	0.054	0.903	8.992	0.0036	0.297	0.979	0.7186	0.231	0.953
	*4*	1.990	0.040	0.851	10.03	0.0013	0.133	0.957	0.6153	-0.39	0.971
	*3*	1.621	0.035	0.901	8.445	0.0013	0.098	0.901	0.4892	-0.34	0.957
	*2*	0.951	0.065	0.978	3.249	0.0463	0.489	0.997	0.2739	0.739	0.798
	*1*	0.489	0.103	0.997	1.675	0.1700	0.477	0.997	0.1395	0.480	0.697
OSMG	*5*	1.992	0.089	0.956	9.938	0.0358	3.544	0.999	0.7862	3.285	0.659
	*4*	1.586	0.085	0.902	8.007	0.0586	3.761	0.999	0.6216	2.839	0.619
	*3*	1.371	0.107	0.881	5.907	0.1170	4.085	0.999	0.4512	2.230	0.580
	*2*	0.912	0.122	0.888	3.850	0.2530	3.751	0.999	0.2892	1.524	0.549
	*1*	0.587	0.219	0.812	1.812	1.0960	3.600	0.999	0.1349	0.746	0.520

**Figure 11:**
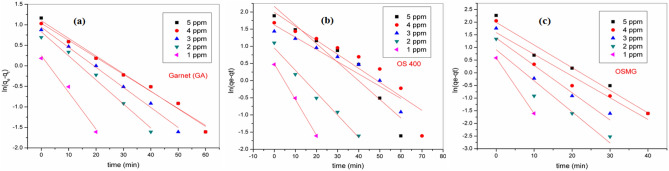
Pseudo-first-order kinetics for adsorption of MB dye onto (**a**) GA, (**b**) OS400 and (**c**) OSMG.

The pseudo-first order equation is defined as follows:

11 $$\mathrm{ln}\left({q}_{e}-{q}_{t}\right)=\mathrm{ln}{q}_{e,1}-{k}_{1}t$$

where qe (mg.g^−1^) is the amount of MB dye adsorbed per unit mass of the sorbent at equilibrium and and q_t_ at a time t (mg.g^−1^).while, K_1_ (min^−1^) is the rate constant of PFO kinetic. However, Fig. 11 reveals the slope and intercept of the log (q_e_-q_t_) vs. time curve, which used to determine the values of the rate constant (k_1_) and equilibrium capacity (${q}_{e,1,\mathrm{cal}})$.

**Figure 12:**
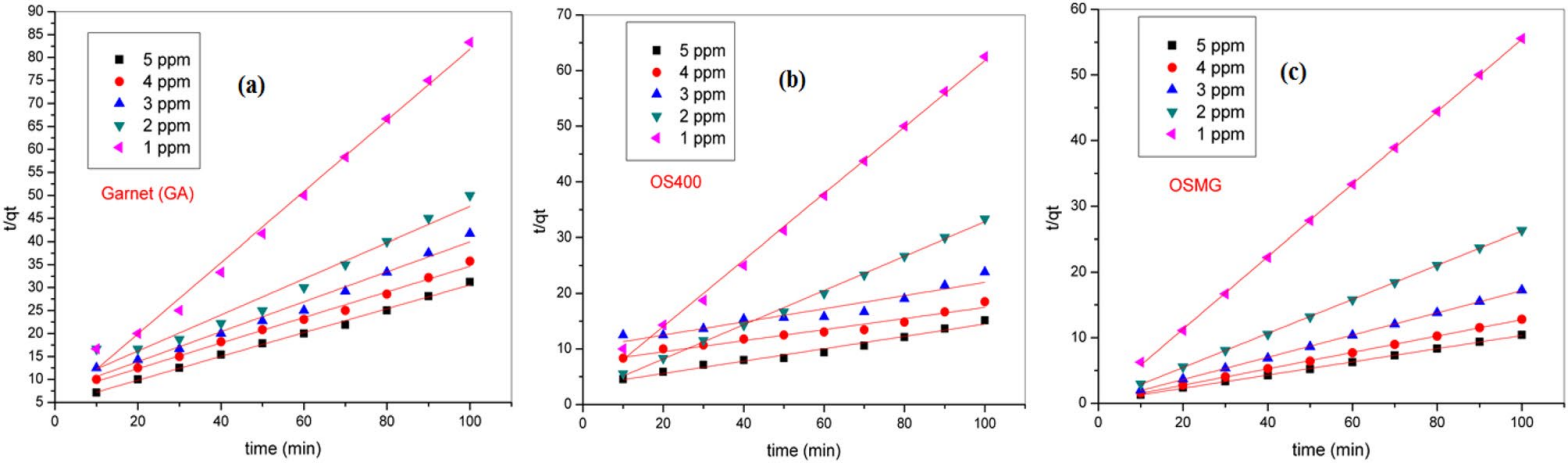
Pseudo-second-order kinetics for adsorption of MB dye onto (**a**) GA, (**b**) OS400 and (**c**) OSMG.

Furthermore, the pseudo-second order kinetic model's equation is expressed as follows:

12 $$\frac{\mathrm{t}}{\mathrm{qt}}=\frac{1}{{\mathrm{k}}_{2}{\mathrm{q}}_{\mathrm{e},2}^{2}}+\frac{1}{{\mathrm{q}}_{\mathrm{e},2}}\mathrm{t}$$

13 $$h={k}_{2}{q}_{e,2}^{2}$$

where *k*_*2*_ (g/mg.min) is the rate constant of PSO model, (mg/g.min). The values of *k*_*2*_ can be calculated using the plot of t/q vs. t as shown in Fig. 12. While (h) is the initial rate of adsorption.

The intra-particle diffusion model was represented by Eq. (14) where *k*_*i*_ is the intra-particle diffusion rate constant (mg/g min^½^) and *C* (mg/g) is the film thickness,

14 $${q}_{t}={K}_{i }{t}^{0.5}+C$$

The results obtained in Table 7 revealed that the highest initial concentration (5 ppm) was considered the best behavior with kinetic data calculated from the pseudo second-order model (correlation coefficient R^2^ ≥ 0.99).

It is noteworthy that the calculated PFO kinetic parameters were also compatible with the experimental data, (R^2^ ≈ 0.90–0.98)^98^. According to the correlation coefficient (R^2^), the PSO model was more suitable than the PFO model, Similar trends have been observed by several authors^46,99,100^. For the most of adsorption systems of dye, a pseudo-second-order model usually provides a better representation of the kinetic adsorption data^86^, and chemisorption is demonstrated as a result which involves electrostatic force and valence forces by sharing or exchanging electrons between the adsorbent and adsorbate^100^.

**Figure 13:**
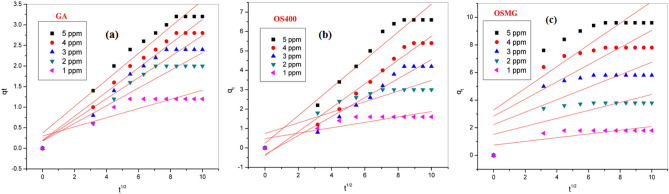
Weber-Morris Intraparticle diffusion model for the adsorption of MB onto (**a**) GA, (**b**) OS400 and (**c**) OSMG.

Figure 13 and Table 7 show the experimental kinetic data using the Weber-Morris model^95^ to explore the intra-particle diffusion process. Moreover, IPD was applied to determine the rate-controlling step of the sorption process^101^.

In the present study, the initial and final stages of adsorption have different mass transfer rates^100^. The deviation from a linear plot due to the resistance provided by the boundary layer which confirms the intra-particle diffusion is not only the rate-controlling step^102^.

### Thermodynamic study

**Figure 14:**
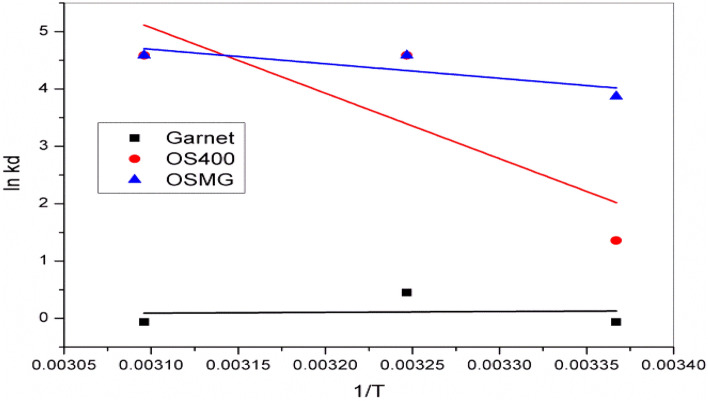
Van’t Hoff thermodynamics model for GA, OS400 and OSMG.

Thermodynamic parameters provide an important role in the behavior of the adsorption process. Figure 14 reveals Van’t Hoff thermodynamics model which was carried out by constructing the reaction at different temperatures between 297 and 323 K. The values of calculated parameters are commonly used to investigate if the adsorption process were endothermic or exothermic, spontaneous or non-spontaneous mechanism.

Thermodynamic parameters are estimated from the equations below:

15 $$\mathrm{ln Kd }= \frac{\Delta {\mathrm{S}}^{\mathrm{o}}}{R} - \frac{\Delta {H}^{o}}{RT}$$

16 $$\mathrm{Kd }= \frac{\mathrm{qe}}{\mathrm{Ce}}$$

17 $$\Delta {G}^{o}=\Delta {H}^{o} - T\Delta {S}^{o}$$

where *q*_*e*_ (mg/g) and *C*_*e*_ (mg/l) are the amount of the dye adsorbed on the composite surface and the residual concentration in the solution at equilibrium (mg/l), respectively; *R* is the universal gas constant (8.314 J mol^−1^ K^−1^); and *T* is the absolute temperature (K)^103,104^. (ΔG◦) kJ/mol is Gibbs free energy, (ΔH◦) (J/mol) enthalpy change, and (ΔS◦) entropy change (J/mol k).

Thermodynamic parameters (ΔH◦), (ΔS◦), and (ΔG◦) have been calculated and tabulated in Table 8. It has been noted that the value of (ΔG◦) was positive at all temperatures which indicates a non-spontaneous process. The results depicted in Table 8 show that the exothermic reaction for (OS400) and (OSMG) is due to a negative value of (ΔH◦)^46,104^. additionally, the positive value in the case of (GA) treatment refers to the endothermic process^105^.

**Table 8:** Thermodynamic parameters for adsorption of MB dye into GA, OS400 and OSMG.

Adsorbent	Temp (K)	*∆G* (kJ/mol)	*∆S* (J/mol k)	*∆H* (KJ/mol)
GA	*297*	1.1294	−0.0023	0.4454
	*308*	1.1547		
	*323*	1.1892		
OS400	*297*	4.1012	−0.2943	−83.2956
	*308*	7.3382		
	*323*	11.7521		
OSMG	*297*	8.2999	−0.0966	−20.3783
	*308*	9.3620		
	*323*	10.8104		

Worthy mentions that, the negative values of entropy (ΔS◦) for MB dye onto (GA), (OS400), and (OSMG) explained the irregularity at the interface of the solid/solution decreases and a less chaotic condition of the adsorbed MB ions on the adsorbent^87,106^.

## Adsorption mechanism

In order to fully grasp the adsorption mechanism by which methylene blue dye attaches to the synthesized adsorbents, a piece of accurate knowledge of the mechanism of adsorption is essential. In fact, the sorption process of MB dye occurs through a combination of chemisorption and physisorption, depending on the surface and textural characteristics of the adsorbent, and the way in which the adsorbate diffuses towards it.

**Figure 15:**
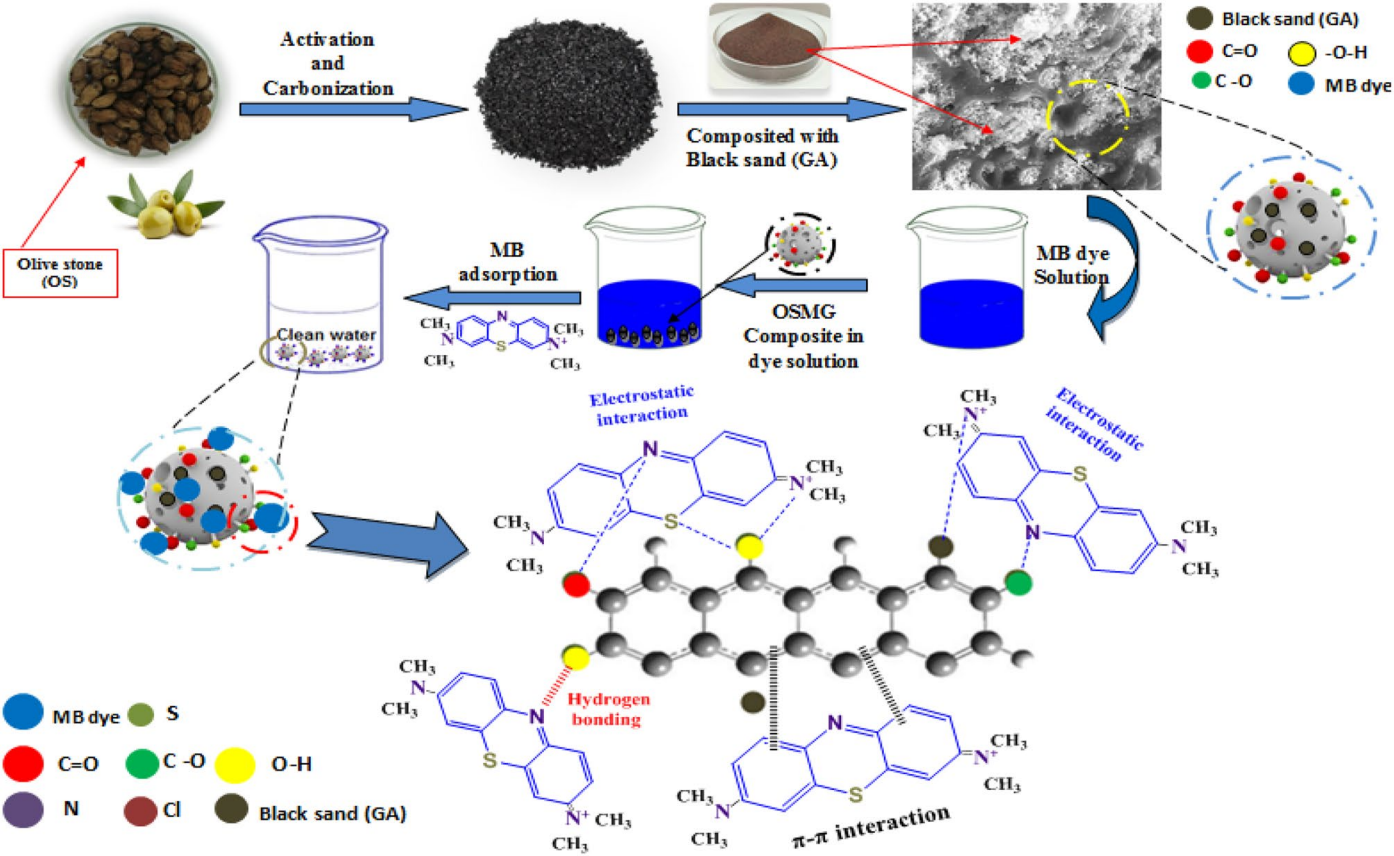
A graphical illustration of the synthesis process for composite adsorbents and proposed adsorption mechanism.

In this particular case, the dye is removed by the oxygenated functional groups (–COO^−^, and –OH) present on the surface of the as-prepared (OSMG) in aqueous media, as evidenced by FTIR spectra. These groups have strong ability to attract the species with a contrary charge and repel those with the same charge. As a result, the cationic nature MB causes it to be strongly attracted from the solution to the surface of (OSMG) composite. fuethermore, the removal of the dye involves electrostatic interaction, hydrogen bonding, and π–π interactions with aromatic rings of the dye, which may also play a vital role in the adsorption mechanism as demonstrated in Fig. 15.

The adsorption process follows the Freundlich isotherm, and the kinetic data suggests that the adsorption process for MB dye is controlled by the chemisorption mechanism. A similar phenomenon has been observed when using Kendu fruit peel as activated carbon^92^.

## Desorption of MB dye and reusability studies

**Figure 16:**
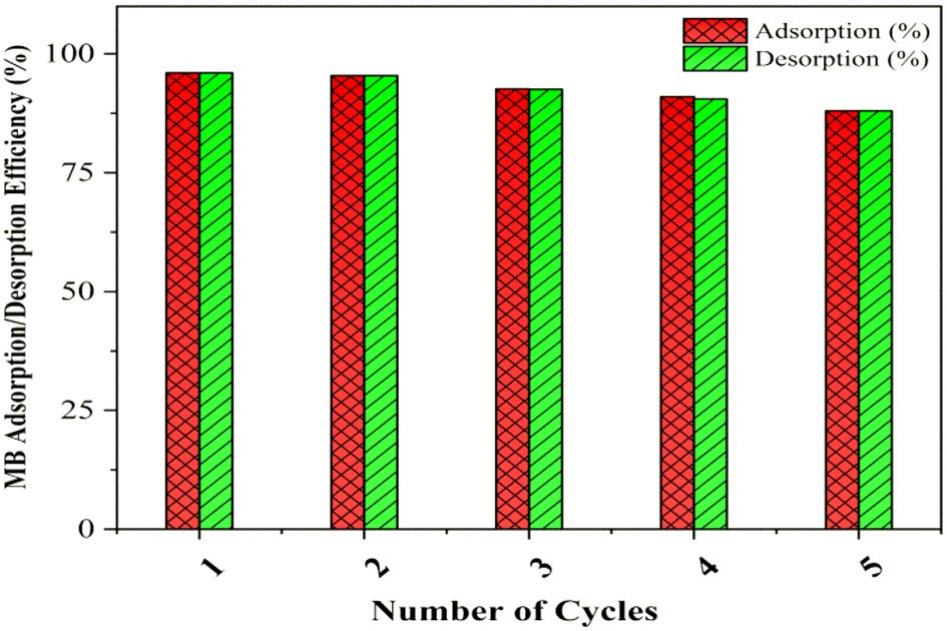
Adsorption–Desorption efficiency of MB onto (OSMG) adsorbent after 5 cycles.

The adsorption and desorption percentages of methylene blue dye were investigated to determine the reusability performance of the (OSMG) adsorbent as demonstrated in Fig. 16.

When in an acidic medium, the active site groups located on the surface of the (OSMG) become protonated, which causes a decrease in the electrostatic attraction between the adsorbate (MB) and the active centers of the adsorbent. This result in the diffusion of MB molecules into the acidic solution. As demonstrated in Fig. 16 sequential adsorption–desorption experiments using 0.1 M nitric acid solution as desorption agent was assessed.

Thus, (OSMG) composite was able to be recycled for five cycles maintaining its high adsorption capacity, with a consistent MB adsorption/desorption ratio, ranging from 97 to 88%. These finding confirm that the (OSMG) composite can be used for water treatment, since it can be effectively and rapidly regenerated without losing its significant adsorption capacity. This behavior has also been reported in previous literatures^107,108^**.**

## Conclusions

This study investigated the synthesis of a novel adsorbent material, OSMG, by impregnating activated carbon (OS400) from olive stones (agro-waste) with Egyptian black sand (GA). The adsorbent’s effectiveness in eliminating methylene blue dye was evaluated. Results revealed that the raw material’s activation at a temperature of 400 °C to produced carbonaceous materials with favorable structural and surface chemistry characteristics. This improved properties enabled the new composite (OSMG) to effectively remove 98% of MB dye under a variety of experimental conditions. Adsorption kinetic and isotherm models showed that the adsorption mechanism primarily involves chemisorption through electrostatic attraction, hydrogen bonding,

and π–π interactions. The Langmuir’s maximum adsorption capacities (Q_max_) of the synthetized (GA), (OS400), and (OSMG) were 41.014, 111.30, and 141.599 mg⋅g^−1^ respectively. The activated carbon derived from olive stone incorporated with garnet could be used at least for five consecutive adsorption/desorption cycles while retaining its adsorptive capacity. These results demonstrate that the synthesized composite is a highly efficient adsorbent that has great performance potential for sustainable use in removing various dyes and the other organic pollutants from industrial effluents.

## Data Availability

The data used to support the findings of this study are available from the corresponding author upon request.
